# Supplementary material for: Emergent interface vibrational structure of oxide superlattices
Source: Nature. 2022 Jan 26;601(7894):556–61. doi: 10.1038/s41586-021-04238-z (PMC8791828; doi:10.1038/s41586-021-04238-z)
Supplement: Supplementary file 1 — This file contains Supplementary Sections 1–6, including Supplementary Tables 1–3, Figs. 1–17 and References. [file 41586_2021_4238_MOESM1_ESM.pdf]

nature portfolio

https://doi.org/10.1038/s41586-021-04238-z

Supplementary information

Emergent interface vibrational structure of oxide superlattices

In the format provided by the
authors and unedited

Supplemental Information:
Emergent Interface Vibrational Structure of Oxide Superlattices

Eric R. Hoglund[1*], De-Liang Bao[2], Andrew O'Hara[2], Sara Makarem[1], Zachary T. Piontkowski[3], Joseph R. Matson[4], Ajay K. Yadav[5], Ryan C. Haisimaier[6], Roman Engel-Herbert[7,8], Jon F. Ihlefeld[1], Jayakanth Ravichandran[9], Ramamoorthy Ramesh[5], Joshua D. Caldwell[4], Thomas E. Beechem[3,10,11], John A. Tomko[12], Jordan A. Hachtel[13♠], Sokrates T. Pantelides[2,14+], Patrick E. Hopkins[1,12,15○], and James M. Howe[1♦]

[1]Dept. of Materials Science and Engineering, University of Virginia, Charlottesville, VA 22904, USA
[2] Dept. of Physics and Astronomy, Vanderbilt University, Nashville, TN 37235 USA
[3] Sandia National Laboratories, Albuquerque, NM 87123, USA
[4] Dept. of Mechanical Engineering and Electrical Engineering, Vanderbilt University, Nashville TN 37235, USA
[5] Dept. of Materials Science and Engineering, University of California Berkley, Berkley, CA 94720, USA
[6] Dept. of Materials Science and Engineering, Pennsylvania State University, University Park, PA, 16802, USA
[7] Paul-Drude-Institut fur Festkörperelektronik, Hausvogteiplatz 5-7, 10117 Berlin, Germany
[8] Institut für Physik, Humboldt-Universität zu Berlin, Newtonstrasse 15, D-12489 Berlin, Germany
[9] Dept. of Chemical Engineering and Materials Science, University of Southern California, Los Angeles, CA 90089, USA
[10] Center for Integrated Nanotechnologies, Sandia National Laboratories, Albuquerque, NM, 87123
[11] School of Mechanical Engineering and the Birck Nanotechnology Center, Purdue University, West Lafayette, Indiana 47907, USA
[12] Dept. of Mechanical and Aerospace Engineering, University of Virginia, Charlottesville, VA 22904, USA
[13] Center for Nanophase Materials Sciences, Oak Ridge National Laboratory, Oak Ridge, TN 37830, USA.
[14] Dept. of Electrical and Computer Engineering, Vanderbilt University, Nashville TN 37235, USA
[15] Dept. of Physics, University of Virginia, Charlottesville, VA 22904, USA

*erh3cq@virginia.edu
♠hachtelja@ornl.govhachtelja@ornl.gov
+pantelides@vanderbilt.edu
°phopkins@virginia.edu
♦jh9s@virginia.edu

S1 Supplemental Information: Diffraction

The discussion of SADPs shown in Figure 1(b-d) of the main text detailed changes in the orientation of the *Pbnm* unit-cell that results from octahedral tilting, and further implicates that the octahedral tilting might also be what mediates the changes in orientation. In this supplemental section, simulations are presented that support and clarify ordered reflections in the SADPs, lattice parameters for the superlattices are quantified, and other features in the diffraction pattern that may implicate changes in structure or phonon softening will be discussed further. Lastly, scanning convergent-beam electron diffraction is used to spatially map octahedral tilting as a function of position, which is like the SADPs but includes spatial dimensions in addition to scattering dimensions.

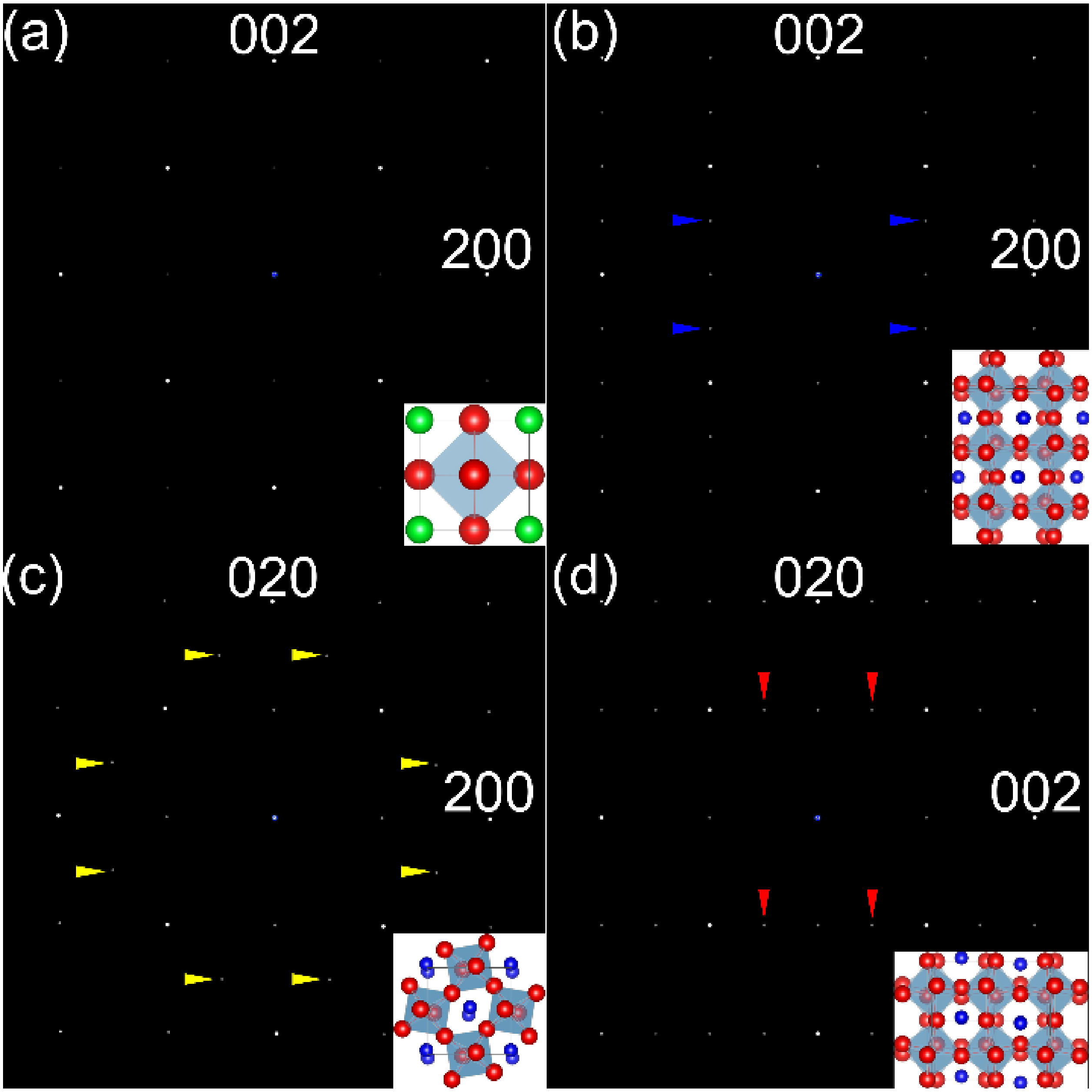

Figure S1. Simulated SADP for: (a) $Pm\bar{3}m$ and (b,d) *Pbnm* along the out-of-phase tilt axis and (c) along the in-phase tilt axes. Blue, red, and yellow arrows indicate the same ½{201}$_{pc}$, ½{021}$_{pc}$, and ½{130}$_{pc}$ ordered reflections, respectively, observed in the experimental SADP shown in Figure 1. Note that here the ordered reflections have different Miller indices that are based on the *Pbnm* pseudo-cubic axes while in Figure 1 the ordered reflections are labeled based on the STO axes aligned with the film geometry.

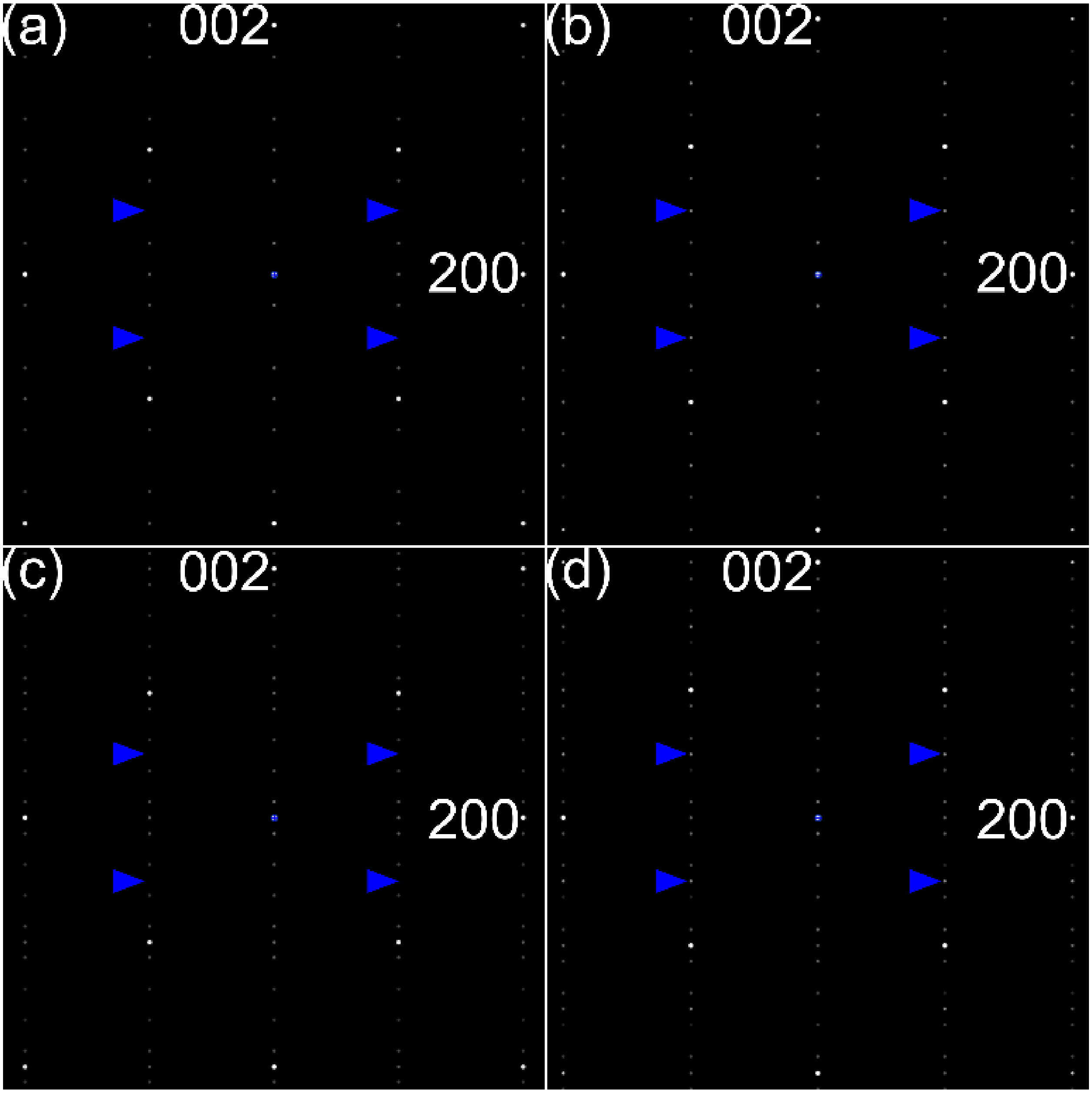

Figure S2. Simulated SADP for SL2 constructed form (a) four *Pm*$\bar{3}$*m* and (b) two *Pbnm* unit-cells and SL4 constructed from (c) eight *Pm*$\bar{3}$*m* and (d) four *Pbnm* unit-cells. Blue arrows indicate the absence of ordered reflections in *Pm*$\bar{3}$*m* diffraction patterns and presence of ½$\{201\}_{pc}$ type ordered reflection in *Pbnm* diffraction patterns.

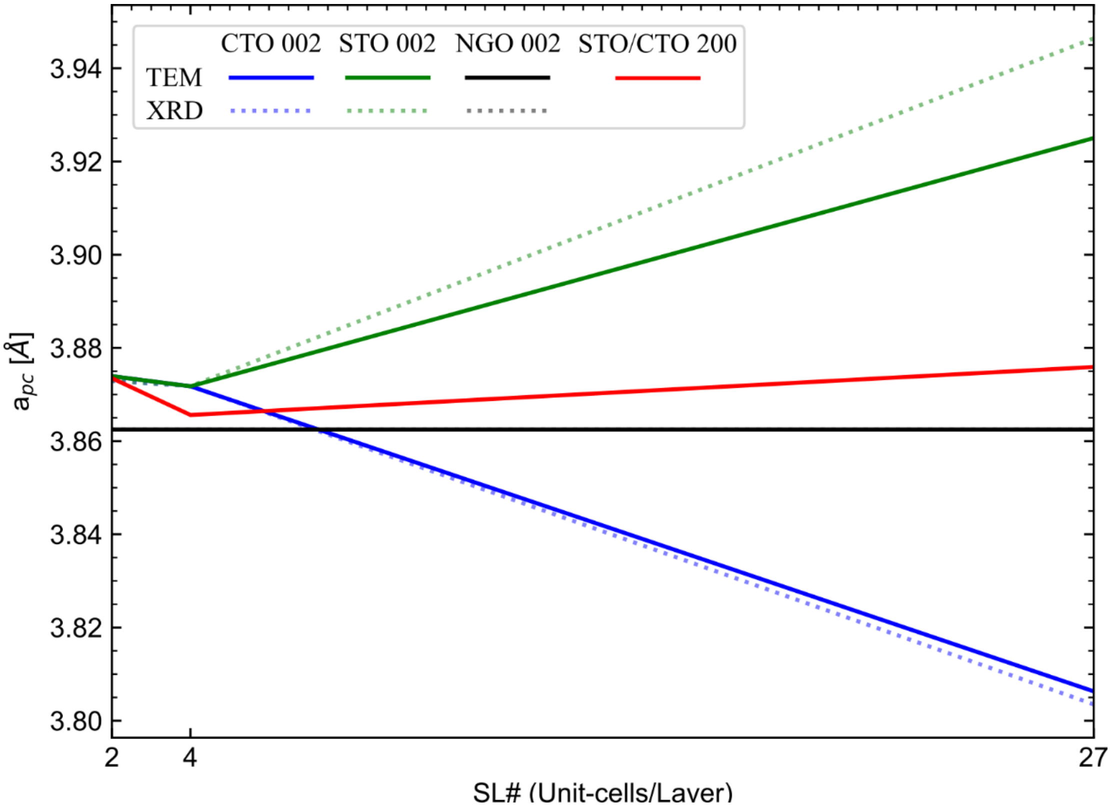

Figure S3. Calibrated lattice parameter calculated from the $(002)_{pc}$ Bragg peak using TEM SADP (solid) and x-ray diffraction (dotted) for CTO (blue), STO (green), and the NGO substrate (black). The in-plane lattice parameter for STO and CTO calculated from the $(200)_{pc}$ Bragg peak in a SADP is shown (red).

XRD was performed with a 2θ-ω scan geometry to determine the out-of-plane pseudo-cubic lattice constant. The NGO substrates $(002)_{pc}$ was used for alignment and the calculated lattice parameter showed minimal deviation between samples. It is assumed that the measured lattice parameter of the NGO substrate should be identical for all techniques and all samples. Therefore, all SADP and XRD measurements of STO, CTO, and NGO were scaled such that the NGO $(002)_{pc}$ lattice parameter equaled the average NGO $(002)_{pc}$ lattice parameter of the XRD scans. Lattice parameters were extracted from the SADP in Figure 1, and the scaled values are shown in Table S1. The lattice parameters calculated from XRD experiments are shown in Table S2. The

scaled lattice parameters from SADP and XRD are plotted together as a function of SL period in Figure S3.

Table S1. Scaled lattice parameters calculated from SADP in SL2, SL4, and SL27. Values are in Å.

	002			200
**SL#**	**STO**	**CTO**	**NGO**	**STO/CTO**
**2**	3.874		3.862	3.873
**4**	3.872			3.866
**27**	3.925	3.806		3.876

Table S2. Scaled lattice parameters calculated from XRD in SL2, SL4, and SL27. Values are in Å.

SL#	STO	CTO	NGO
**2**	3.873		3.862
**4**	3.872		
**27**	3.804	3.946	

From the measured lattice parameters, it was found that the out-of-plane lattice parameter converged to a single STO-CTO value that was approximately the average of the two unrelaxed lattice parameters of SL27. This is the general theme observed in all crystallographic and vibrational structure analysis herein, that is, as the number of unit-cells in a layer decreases the structure converges to a singular, uniform, intermediate structure. The in-plane lattice parameter is different (but nearly equivalent) to the substrate in SL4 and 27. SL2 had in-plane lattice parameters like their out-of-plane lattice parameter and were larger than SL 4 and 27, indicating that the film in-plane lattice parameters are not determined by the substrate.

In the SADPs shown in Figure 1(b-d) a rich set of information is present between the Bragg peaks.[2] In SL27 shown in Figure 1(b), closely spaced superlattice reflections extend from the Bragg peaks making them look streaked. The same superlattice reflection are seen in SL4 and

SL2, but are much further spaced and appear as distinct peaks because of the much larger real-space periodicities. An interesting diffuse background intensity is found in SL4 at the location of the ordered reflections. We will speculate to their origin but defer to future research to ascertain the true origin of the diffuse intensity. Diffuse intensity in the background of diffraction patterns is associated with the loss of long rand coherence, whether it be from structural static disorder or thermal vibrations. In the present case, we find that the superlattices contain a high degree of long-range static ordering, as clear from the sharp fundamental, ordered, and superlattice Bragg reflections. This remaining option is thermal vibrations. In many phase transitions a phonon mode at a specific momentum vector causes the transition to a lower symmetry structure. In these cases the thermal diffuse scattering, typically considered as a uniform Gaussian like distribution centered at $\boldsymbol{q}$=(000), can become non-uniform and slowly develop into Bragg peaks as the mode softens and forms new Brillouin zones. [3–5] In the case of SL4 we observe with iDPC that octahedral tilting is present throughout the entire superlattice structure, including within STO layers where no natural tilts are present at ambient conditions. SL4 also has the smallest number of unit-cells per layer where the layers can be well defined, see section S2 and the main text for elaboration. From the continuous change in tilting, it is expected that the phonon associated with the tilting is neither completely hardened or softened through the entirety of the structure and may therefore produce a non-uniform thermal diffuse background.

Convergent-beam electron diffraction patterns were acquired for each pixel of a line scan, known as scanning convergent-beam electron diffraction (SCBED), across the STO-CTO interfaces. Ordered Laue zones in the atomic resolution SCBED allows mapping of octahedral tilt.[6,7] Line scans of a two, four, and twenty-seven unit-cell superlattice are shown in Figure S4.

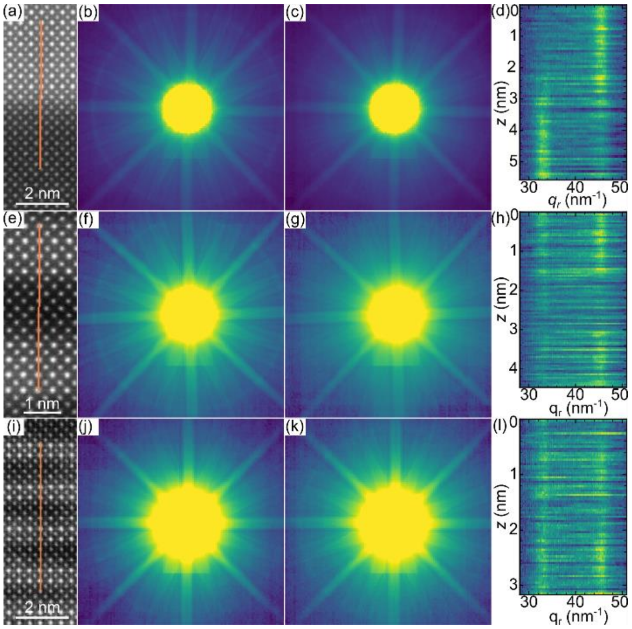

Figure S4. Scanning-CBED measurements from (a-d) SL27, (e-h) SL4, and (i-l) SL2. The position of line scans is indicated in the (a,e,i) ADF reference image. The PACBED pattern from the STO and CTO layers are shown in (b,f,j) and (c,g,k), respectively, show layer dependent fundamental and ordered Laue zones. (d,h,l) Background subtracted radially integrate CBED patterns show the existence of ordered reflections, indicative of octahedral tilting, as a function of position as the probe scanned across the superlattices.

The abrupt change in contrast of A-site atoms in the high-angle annular dark-field ((HA)ADF) signal shows the chemical abruptness of the superlattices. To visualize the relative difference in diffraction patterns taken from STO and CTO layers we compare the position averaged CBED (PACBED) from each layer, as shown in Figure S4(b,c). In the large period twenty-seven unit-cell superlattice the STO PACBED had a Laue zone corresponding to the intersection of the Ewald sphere with fundamental Bragg reflections of the $Pm\bar{3}m$ reciprocal lattice. The CTO PACBED had an identical fundamental Laue zone and an additional Laue zone, with half the radius of the fundamental, corresponding to the intersection of the Ewald sphere with half-integer ordered reflections appearing from TiO_6 tilting. The existence of tilting in CTO and not in STO further supports the conclusion from SAED that STO and CTO are relaxed to their monolithic phases. When the number of unit-cells in a layer is reduced to four per layer the

intensity of fundamental and ordered Laue zones in the CTO PACBED were much more similar, as shown in Figure S4(g). This could indicate some reduction in the octahedral tilt-angle, but in general shows that octahedral tilting still exists in CTO as expected from the SAED. With the decrease in layer thickness the STO PACBED now shows both a fundamental and ordered Laue zone much like CTO, as shown in Figure S4(f). Therefore, we conclude that the STO has inherited TiO_6 tilting from the CTO layers because of the reduced layer thickness. The inheritance of octahedral tilting persist with a further reduction to two unit-cells per layer and the comparable intensity of ordered and fundamental Laue zones is seen in both layers. The observed ordered reflections show that the STO layers are inheriting octahedral tilting and becoming adapting to the CTO crystal structure.

We know that the layers are changing and wanted to investigate more local changes. By radially integrating the CBED and subtracting a background power law the relative intensity of ordered and fundamental reflections at each pixel of a line scan is compared, as shown in Figure S4(d,h,l). In SL27 the intensity of the fundamental Laue zone is uniform in both CTO and STO layers and the intensity of the ordered Laue zone is uniform in the CTO layer showing that the structure of the layers is uniform, as shown in Figure S4(d). At the STO-CTO interface a transition region occurs over three atomic planes. In this transition region an ordered Laue zone appears in STO and gradually increases intensity as the probe entered the CTO. There is therefore a chemically abrupt and structurally diffuse interface that results from the coupling of STO and CTO layers, much like $La_{0.5}Sr_{0.5}TiO_3$/CTO and $NdGaO_3$/CTO interfaces.[8] When the number of unit-cells is decreased to four or two unit-cells per layer, where the structurally defined interface size is the same size as the chemically defined layers and octahedral tilting is present everywhere, the distinction between an interface region becomes less discernable. This

could, in-part, be from relatively small contrast changes buried in noise, but implicates that the changes in ordered Laue zone intensity, indicating inheritance of octahedral tilting in STO and reduction of titling in CTO is from the overlapping structurally diffuse interfaces and the superlattice is transitioning to a system structurally defined by such interfaces.

S2 Supplemental Information: ADF and iDPC

In the STO layer of SL27 an in-phase tilt-angle of 1.84° and 2.69° STO was measured in the TiO_2 and AO planes, respectively, since no tilting is present and represents a bound to measurement error. In CTO out-of-phase tilt-angles of 10.04° and 10.32° in the TiO_2 and AO planes, respectively. In SL4 the quantified out-of-phase tilt-angle profile was sinusoidal with an average of 6.34° and 7.39° in the TiO_2 and AO planes, respectively, demonstrating that the layers tilts have accommodated to approach the mean or interface value observed in the larger-period SL27. In SL2 the tilt profile did not have any systematic tilt-angle related to the layer periodicity appearing "dephased" with a nearly constant tilt-angles of 7.138° and 6.096° in the AO and TiO_2 planes, respectively.

The relative proportion of layers and interfaces can be quantitatively assessed by comparing the volume fraction of interfaces, STO, and CTO defined by

$$V_{int} = \frac{1}{n-1} \tag{1a}$$

$$V_{STO} = V_{CTO} = \frac{n-2}{2(n-1)} \tag{1b}$$

In a large-period superlattice *n* is large so $V_{ATiO_3} \gg V_{Int}$. For a four unit-cell superlattice $V_{ATiO_3} = V_{Int}$ such that the interface is of equal importance to still existing layers. In the extreme

of the two unit-cell superlattice V_{ATiO_3}=0 and V_{Int}=1 such that the entire superlattice is an ordered structure.

S3 Supplemental Information: DFT

In SL8 there is an asymmetry in tilt-angle gradient at the interface. The coupling extends further into the STO layer than into the CTO layer. The STO tilt-angle is also non-zero, unlike in bulk STO. The preferential increase in STO tilt-angles, relative to decreasing CTO angles, suggests that the O-Ti-O tilt mode is softer in STO. We cannot entirely rule out the influence of strain on this behavior.

Relaxed structures have lattice parameters different than the bulk STO and CTO lattice parameters. In SL8 the structure did not relax to the bulk like lattice parameters in the CTO and STO layers. A 1.8% compressive strain and 1.7% tensile strain is present in the STO and CTO layers, respectively, relative to bulk like phases.

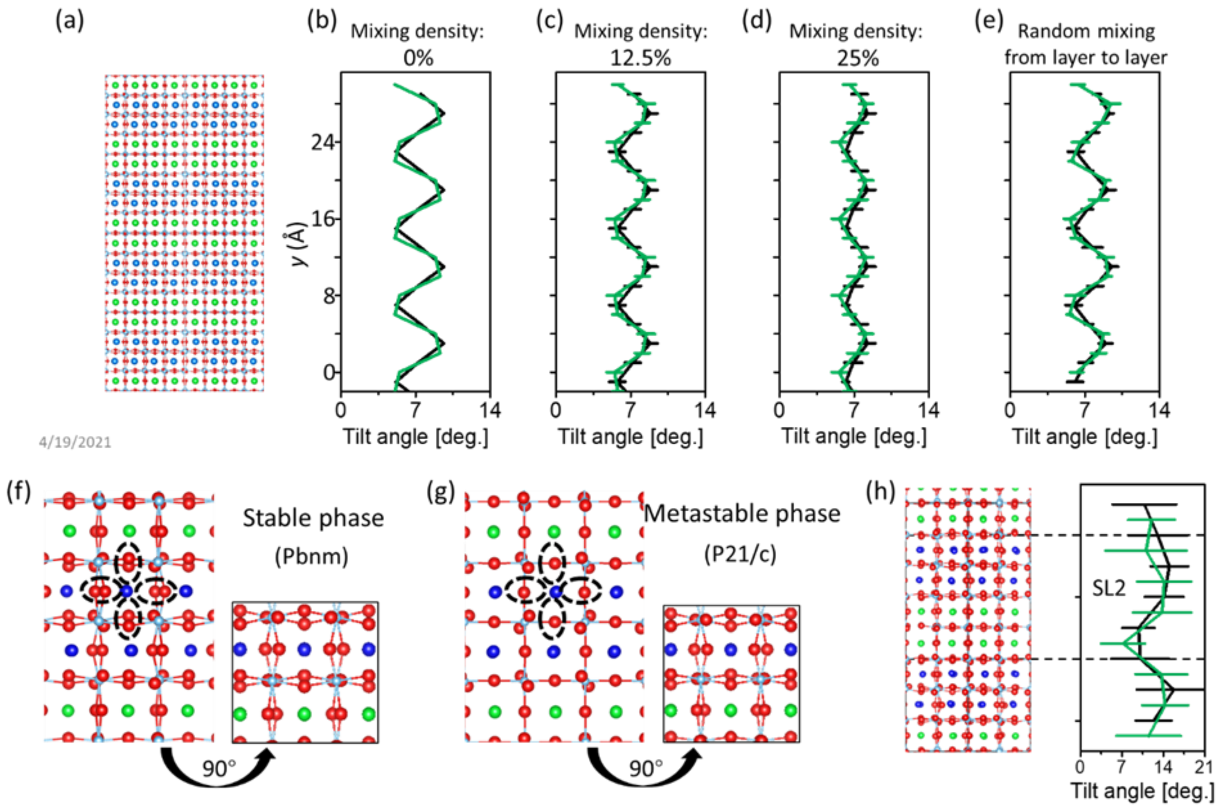

Figure S5. (a) Atomic model of SL2. (b-d) O-Ti-O tilt-angles as the uniform intermixing density changes from 0% to 25%. The y-axis scales are the same as in (a).). (e) O-Ti-O tilt-angles when a 14.1% intermixing density is distributed nonuniformly in the layers. (f) Stable *Pbnm* phase of SL2. (g) Metastable phase of SL2 with *P21/c* symmetry. (h) O-Ti-O tilt-angles when oxygen vacancies are present, distributed randomly in the layers, with an overall density of 2.2x10^{21} cm^{-3} (this is a high density of vacancies, limited by computational costs)

In Figure 1, it is noteworthy in that the calculations show distinguishable oscillations between octahedral tilts in the STO and CTO layers in SL2, whereas the experimental angles appear decoupled from the chemical identity of the planes. This may be caused by finite amounts of intermixing across the interface (Figure S5(a-e)), which is difficult to prevent experimentally, or a metastable phase (Figure S5(g)). Tilt-angles with different intermixing densities are calculated and shown in Figure S5(b-e). As the uniform intermixing density goes up, the tilt-angles are greatly decreased to an average value of 7°. Meanwhile, if the intermixing density is nonuniform (which is more likely experimentally), the tilt-angles not only decrease but also show a

dephasing (Figure S5(e)). The narrowing of the oscillation that is caused by intermixing in Figure S5 is an indication that intermixing and/or thermal effects that can occur at room temperature may be responsible for the absence of oscillations in the experimental data of Figure 1(m). DFT calculations predict that SL2 may have a novel metastable phase with *C2/m* symmetry. Comparing with the stable *P21/c* phase, the metastable phase brings tiny lattices change to the crystal, namely a compression of 0.35% in *a*, and a stretch of 0.35% and 0.1% in *b* and *c*, respectively, and an increased total energy of 1.85 meV/atom. The small energy increase and relationship between the structural space groups suggests that the existence of the metastable phase is highly possible, particularly at elevated temperatures. For the metastable phase, as shown in Figure S5(g), along the [110] direction the horizontal and vertical O-Ti-O tilt-angles are zero for the higher symmetry-phase SL2, while along the [$1\bar{1}0$] the tilts in the low-symmetry phase are still present. In such a case, measuring experimentally at a specific direction would obtain smaller tilt-angles than the expectation based on the stable state. The presence of rotational domains could lead to an averaging effect. All the above suggests that the presence of a temperature-dependent metastable phase and a small amount of intermixing may account for the constant angles in the experimental results of SL2. Moreover, oxygen vacancies are also considered in theoretical calculations. As shown in Figure S5(h), the existence of oxygen vacancies with a density of 2.2x 10^{21} cm^{-3} cause the O-Ti-O tilt angles to increase significantly to over 14°, which is not the case with the experimental results. This result excludes the influence of oxygen vacancies in the experiments and confirms the high quality of the samples.

Additional discussion of Figure 3(b): Since the lattice parameters are optimized for the interfaces in the large-period calculations (e.g., SL8), there is always some strain on the STO and CTO layers. In our averaging method, we find very good agreement between the calculated SL27

phonon DOS and the experimentally measured EELS phonon spectra for SL27. By comparing the black curves with the orange curves in each model, we see that as the layer thickness decreases, the phonon DOS converges towards that of the interface curve, demonstrating the dominance of the interface in thinner layer superlattices. We also note that the mode energies of the three peaks discussed in Figure 3, have energies that correlate with Slater- and Axe-type displacements of the TiO_6 octahedra for a range of perovskites.[9] We also show a visualization of select modes in Figure S6 and Figure S8(b,c).

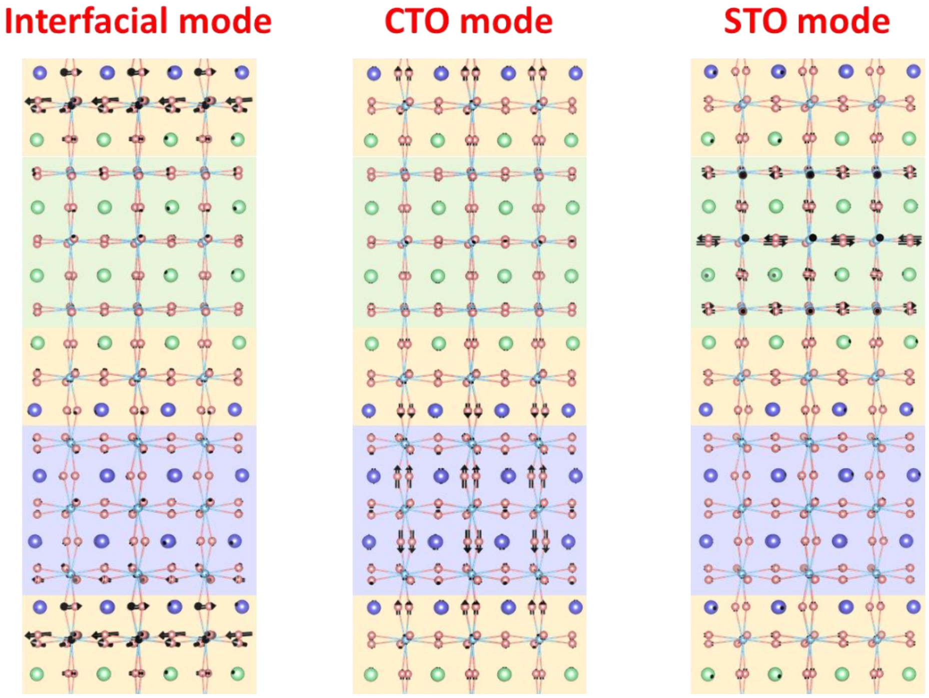

Figure S6. Eigenvectors for interface, CTO, and STO modes in SL4.

The eigenvectors for three typical modes (***q***=0), i.e., an interface mode, a CTO-layer mode, and a STO-layer mode are shown in Figure S6 to emphasize their spatial extent. The three phonon modes are located at 72.3, 90.9, and 70.8 meV. These modes were not selected to correspond to specific peaks in the projected phonon DOS, but to visualize the spatial localization of

vibrations, which does not exist in uniform bulk materials. The vectors can be envisioned as displacement vectors at one moment of time during vibrations. We find that the interface modes are localized to the three-atomic-plane-wide structurally diffuse interface. The STO and CTO layer modes are likewise localized within the layers. We can therefore use DFT to visualize the localization observed in vibrational EELS.

The phonon DOS discussed in the main text is Ti and O projected. This was done to emphasize the impact of octahedral rotations on the phonon DOS and because the EELS analysis energy range was >30 meV. For completeness, we show the remaining A-site projected phonon DOS (PPDOS) in Figure S7. The Sr peak is lower energy than the Ca peak, because Sr is heavier. The Ca+Sr curve in SL2 and SL4 are very similar suggesting that their A-site vibrational response is similar. Most importantly for our justification to neglect A-site bonding, thus emphasizing the impact of octahedral rotation, is that all A-site vibrational responses are less than 30 meV. The A-site vibrations only provide a continuously decaying background to the DOS above 30 meV.

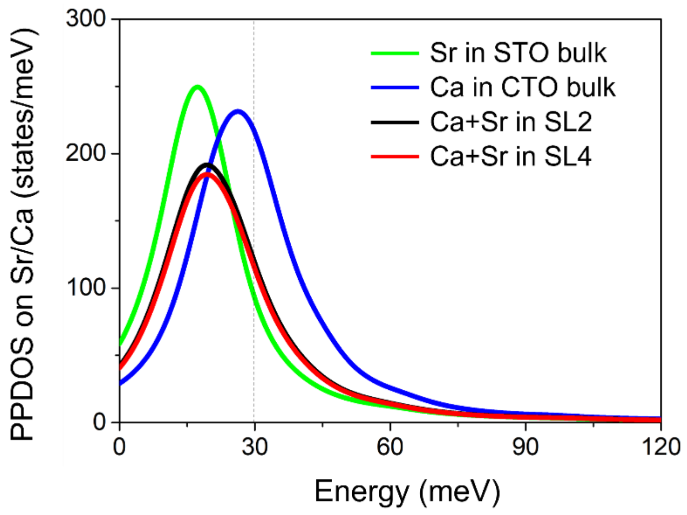

Figure S7. A-site projected phonon DOS.

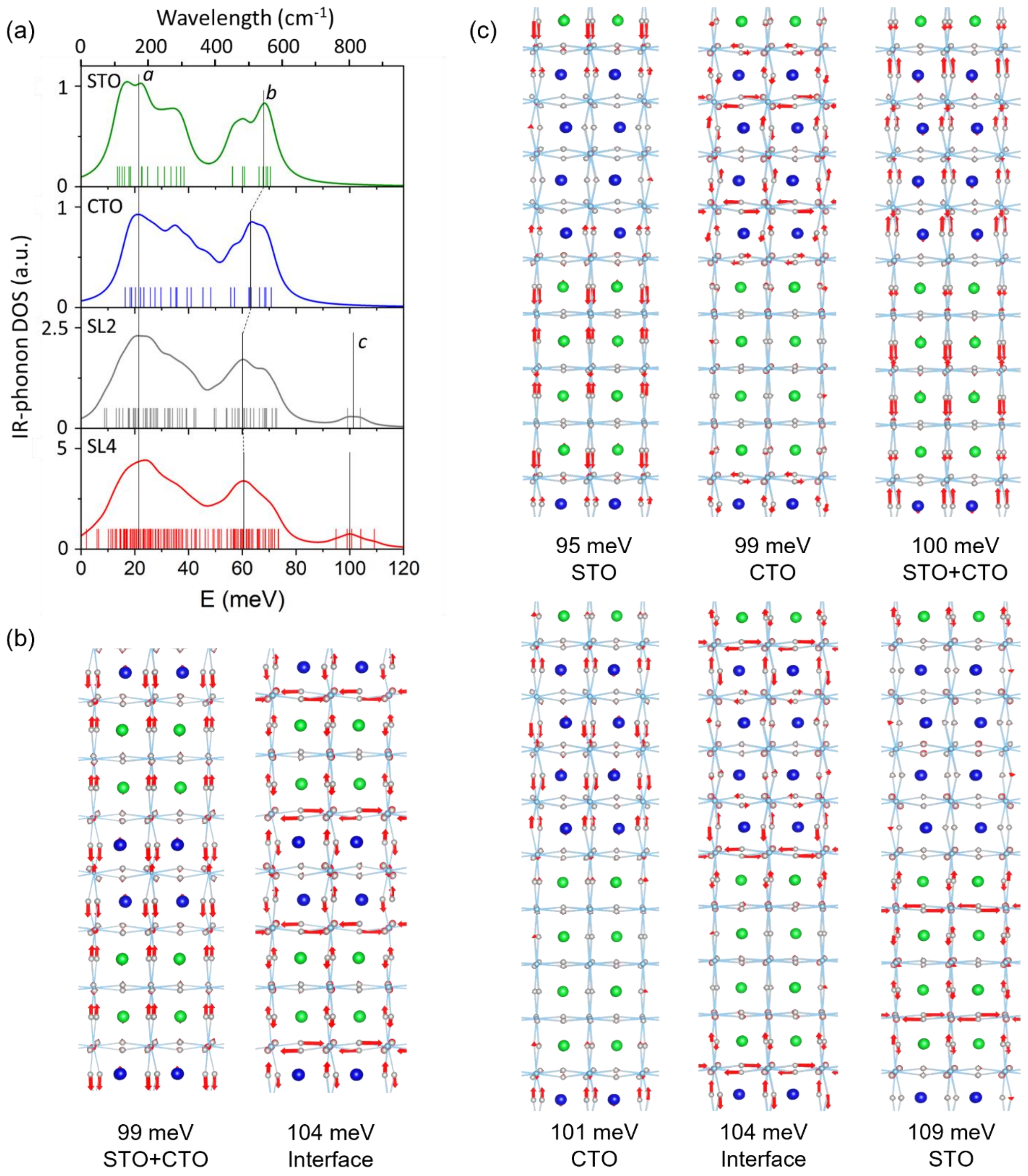

Figure S8. **Infrared active phonon DOS and eigenvectors showing emergent optically active vibrational response in superlattices.** (a) IR-phonon DOS SL4 and SL2 showing the emergence of IR active high-energy vibrations that are not active in either STO or CTO. Eigenvectors of vibrational displacements in (b) SL2 and (c) SL4 showing layer and interface IR active modes.

As a demonstration of the impact of the present work on understanding emergent properties that derive from structural and vibrational features of the SLs, we show in Figure S8(a) the predicted IR-active-phonon DOS for the bulk STO and CTO and the IR-active-phonon DOS for SL2 and SL4. The IR-active-phonon DOS can be compared to the experimental FTIR spectra shown in Figure 4, Figure S13, Figure S15, and Figure S16. We note emergent IR-active phonon modes at surprisingly high energies around 100 meV and also at very low energies. The emergent IR-active phonon modes consist of Ti-O modes localized within STO and CTO layers, delocalized between STO+CTO layers, and localized to interfaces. The density of the latter increases with increasing layer length. These emergent phonon modes are likely to underpin emergent properties, e.g., IR absorption and Raman spectra. Since in complex oxides, structure (e.g., octahedral tilts) and phonons are strongly coupled to electronic and magnetic properties, knowledge of the emergent phonons would help engineer novel properties. In particular, the displacement vectors of the high-energy modes have intriguing localization properties, either at the interfacial TiO_2 planes or within the layers that can potentially host unique spin structures.

S4 Supplemental Information: Vibrational EELS

ADF signal was collected simultaneously with EELS data was used to identify the position of STO and CTO layers and their interfaces. This was done by finding the inflection points in ADF signals via differentiation of the signal, peak finding, then manually removing irrelevant

positions. An example of interface assignment for an off-axis geometry acquired from SL4 is shown in Figure S9.

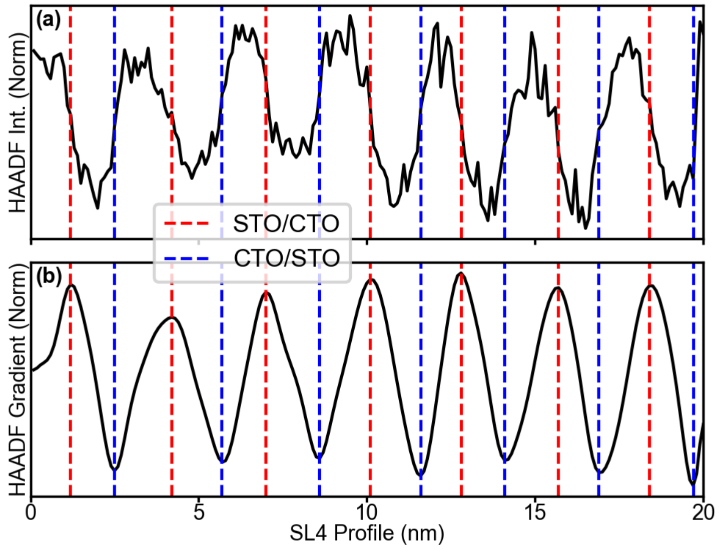

Figure S9. Example of interface assignment in SL4 off-axis signal.

In off-axis EELS line scans, ADF signals are asymmetric with Bragg reflections projecting onto the detector resulting in strain and diffraction contrast, which causes peaks in the ADF signal. An initial concern of qualitatively comparing the vibrational response of the layers and interfaces was that spatial differences in the differential scattering cross-section (with respect to **q**) could easily be confused with shifting peaks leading to a misinterpretation of localized interface modes. One way to determine if the apparent changes are from a change in scattering cross-section or peak energy is to directly compare the spectra. The signal in each layer was averaged into a position averaged energy-loss spectra (PAELS) to reduce the number of spectra to compare and aid interpretation, as shown in Figure S10.

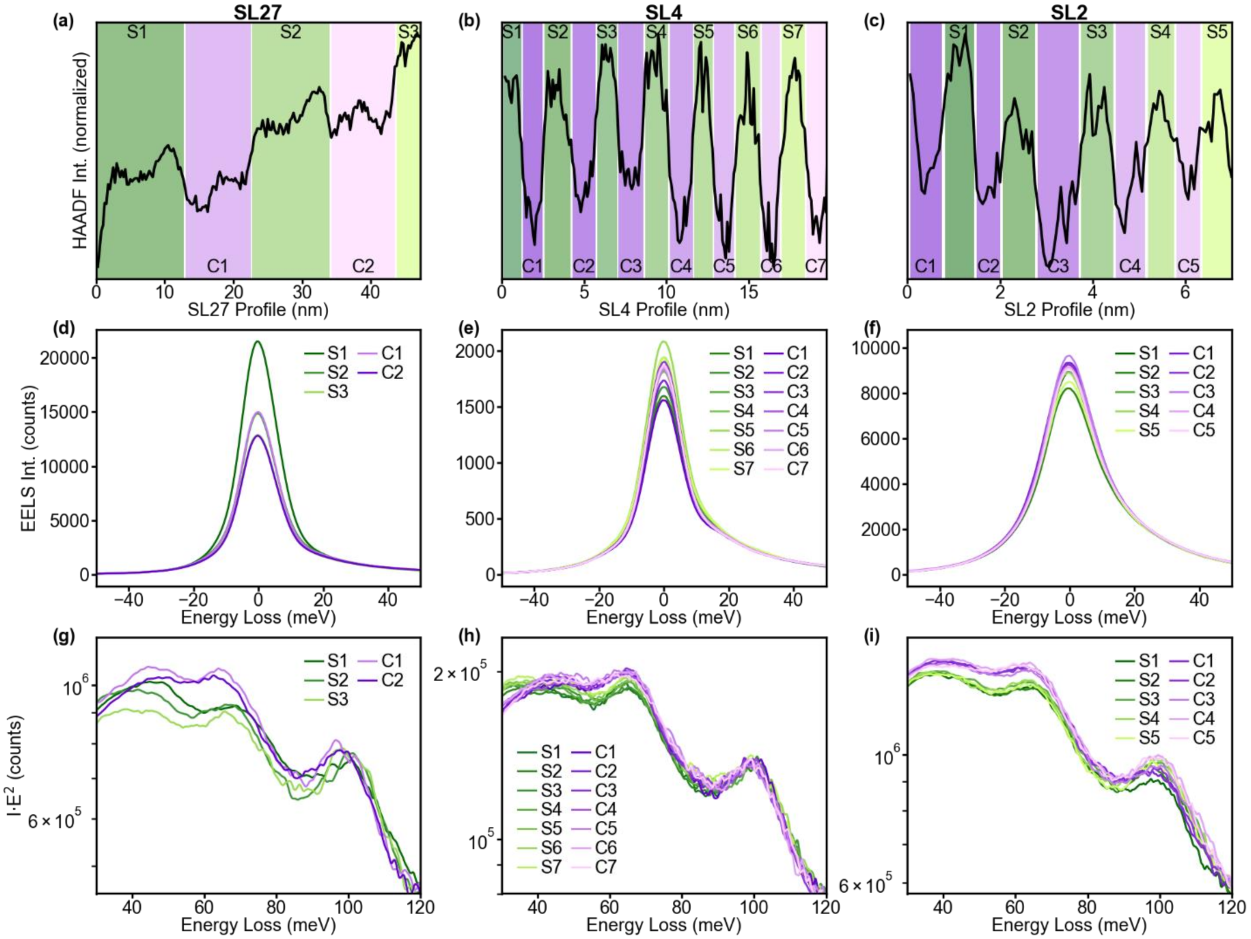

Figure S10. (a-c) ADF signal during the line scan with marker signifying defined layers in the off-axis geometry of (a,d,g) SL27, (b,e,h) SL4, and (c,f,i) SL2. PAELS of the (d-f) quasi-elastic peak and (g-i) phonon-loss region.

The quasi-elastic peak of the PAELS centered at *E*=0 meV is lowest when in vacuum for the off-axis geometry because localized quasi-elastic excitations are not excited. When the probe enters the material, phonons begin to inelastically scatter the incident electron and the quasi-elastic peak increases in intensity. One would expect that the quasi-elastic peak would then linearly increase with thickness like the ADF signal, which is in-part a result of impact scattered phonons. Instead, the quasi-elastic response decreased with each successive period and each layer in a period had nearly the same magnitude quasi-elastic peak (seen most clearly in the SL27 sample which experiences the greatest thickness variation). The overall decrease in signal

could be attributed increase scattering probability of other inelastic excitations, such as plasmons and core-states. Quickly after the probe is in the material the tails of the quasi-elastic peak converge to similar values allowing for the intensity of peaks on the tail of the quasi-elastic peak to be compared directly without considering the influence of the total inelastic differential cross-section. This is further shown by the vibrational response intensity and energy difference between STO and CTO (for example see the ~65 meV region) and similarity between each CTO or STO layer. Although some changes in the phonon-loss region are still observed in the SL27 sample.

We then include a finite interface width into the PAELS, defined as one ±0.39 nm unit-cell, which is approximately one unit-cell on each side of the interface and contains the structurally diffuse interface measured in iDPC experiments. These newly defined layers and convergence point are used to form the layer average EELS in Figure 3 and Figure S11. Layer-averaged spectra in Figure 3 are shown as larger panels in Figure S11 to emphasize the small differences in peak energies, which are also listed in Table S3.

Table S3. Peak energies identified in the layer-averaged EELS spectra of Figure 3 and Figure S11. All energies are listed in meV.

	**Peak 1**			**Peak 2**			**Peak 3**		
	**STO**	**Int.**	**CTO**	**STO**	**Int.**	**CTO**	**STO**	**Int.**	**CTO**
**SL27**	40.1	45.5	46.2	66.7	66.1	63.3	100.3	98.6	96.8
**SL4**	44.0	46.5	46.1	64.7	64.3	64.7	99.0	99.8	98.6
**SL2**	36.2	37.3	38.5	64.3	63.5	63.1	98.8	98.1	98.8

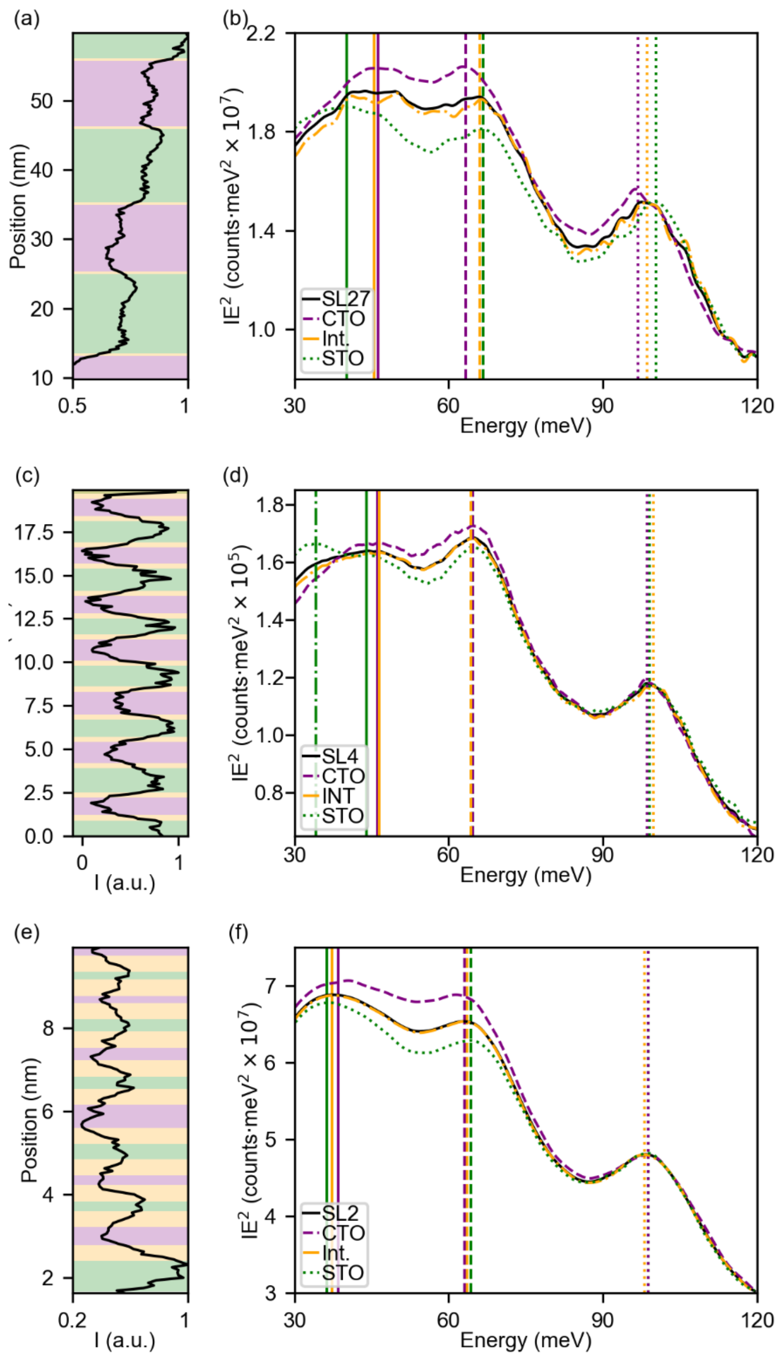

Figure S11. Monochromated STEM-EELS (b,d,f) layer-averaged spectra and (a,c,e) simultaneously acquired ADF signal.

In SL27, the STO, CTO, and interface layers each have unique responses that agree with trends found in DFT experiments. DFT informs that the vibrations in the 30-120 meV energy range are from the Ti and O sublattices, and that the peaks in the Ti+O projected DOS shift with tilt-angle.

We can conclude that the experimental observations are from layer-to-layer changes in TiO_6 tilt. To further emphasize this conclusion, the total and interface spectra can be compared. One would expect the interface spectra to match the total superlattice spectrum if unique interface vibrations are not present because both would be a linear combination of 50% STO and 50% CTO. Instead, there are discrepancies between the interface and total spectra that originate from vibration in the structurally diffuse interface. The small volume fraction of the region ascribed to the interface relative to the large STO and CTO layers suppresses the interface contribution to the total response. See section S2 for discussion regarding the volume fraction of layers. With the discrepancies, and agreement with DFT, we can conclude that the vibrational EELS experiments of SL27 is measuring local changes in vibrations at the interfaces that are a result of an octahedral coupling region. Furthermore, the interface and total spectra are nearly identical when the number of unit-cell per layer is reduced to four, such that the volume fraction of interfaces is identical to the volume fraction of either STO or CTO layers. Now that the interface represents an appreciable portion of the material, the interface vibrational response emerges and contributes appreciably to the total response. The interface contribution to the total vibrations is in addition to the influence that the interface has on the tilts in the STO layers, which will make the STO vibrational response more like the interface response, as also described by DFT.

It is useful to assess the current observations with regard to models for mixed systems, such as the virtual crystal model or two-ion model. [10,11] In the virtual crystal model structural or chemical heterogeneity at interface are incorporated into an interface layer, much like presented in the current work. [10] However, in the virtual crystal model, the interface layer is assigned properties that are effectively represent a disordered combination of the bounding materials. In the present case the interface is not disordered and contains vibrations that cannot be explained by the

bounding materials. The structurally diffuse interface cannot be captured by a virtual crystal because of its unique structural and vibrational state. In a two-phonon model of superlattices phonons are grouped into either coherent or incoherent phonons. [11] The coherent phonons with long wavelengths and mean-free-paths can propagate through the material unscattered, while the incoherent phonons have short wavelengths and mean-free-paths and scatter from the interfaces. In SL27, the two-phonon model may adequately describe the transport of the material with the exception that the scattering probability of some incoherent phonons may not be as large as in a structurally and chemically abrupt interface because the interface structure and vibrations can mediate the transition from one layer to the other. In SL4, the incoherent phonons will also scatter form the interfaces, and there is a much higher density of interfaces so there will be a larger accumulation of scattering probability across the thickness of the superlattice. However, the incorporation of tilt into the STO layers makes the phonon modes more like the CTO modes such that transmission might be increased. In short-period case of SL2, the concept of a two-phonon model need not be considered. The structure in effect no longer has interfaces and the vibrational structure of the entire superlattice is uniform.

Returning to Figure S11, we also observe a fourth peak at 34.1 meV in the STO layer of SL4 but not the CTO/Interface regions. If we compare the position of this peak to the signals in SL27 and SL2 PAELS it can be observed that the SL27 shows enhanced intensity for the STO this same regime with respect to the CTO/Interface, but the SL2 sample does not. Ergo, this is likely indicative of a layer-localized vibrational mode of STO that is absent in the in the "crystal of interfaces" SL2, consistent with the assertion that the octahedral tilts are dominating the measured vibrational differences between the layers. The reason the peak is not directly measurable in the SL27 system is likely a function of thickness, as the SL27 and SL4

acquisitions have highly comparable energy resolution. This is shown in Figure S12, where we measure the full-width at half-maximum (FWHM) of the elastic scattering peak for all three acquisitions. Here, instead of using the full off-axis acquisition we average only the spectrometer channels closest to the optic axis to increase the amount of elastic scattering that is captured in the spectrum. The FWHM of the elastic scattering peak corresponds to the width of the energy band selected by the monochromator and is an accurate measurement of the energy-resolution from the system. For both the SL27 and the SL4 samples we achieve an energy resolution of ~12 meV, and a slightly lower energy resolution (~17 meV) for the SL2 sample. This means the absence of a distinct peak at ~34 meV for the SL27 sample is likely a function of the thickness in the sample rather than the energy-resolution. This is not unexpected, as we previously noted a large thickness variation in the SL27 sample that is not observed in the SL2 or SL4 samples.

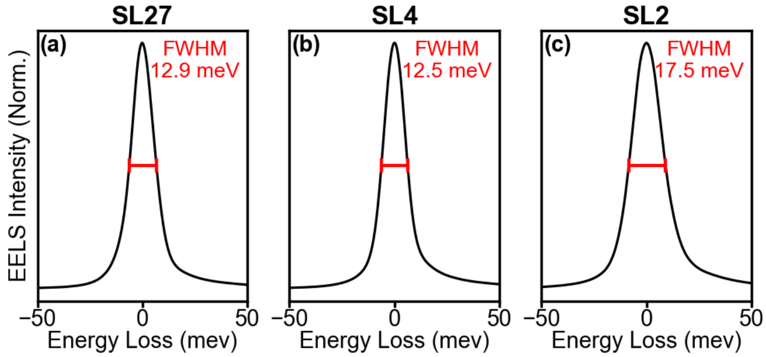

Figure S12. Energy-resolution in each acquisition as measured by the full-width at half-maximum (FWHM) of the elastic scattering peak for each sample.

S5 Supplemental Information: UV-Raman and FTIR

Complimentary UV-Raman and FTIR spectra were acquired to show the response as a function of interface density.

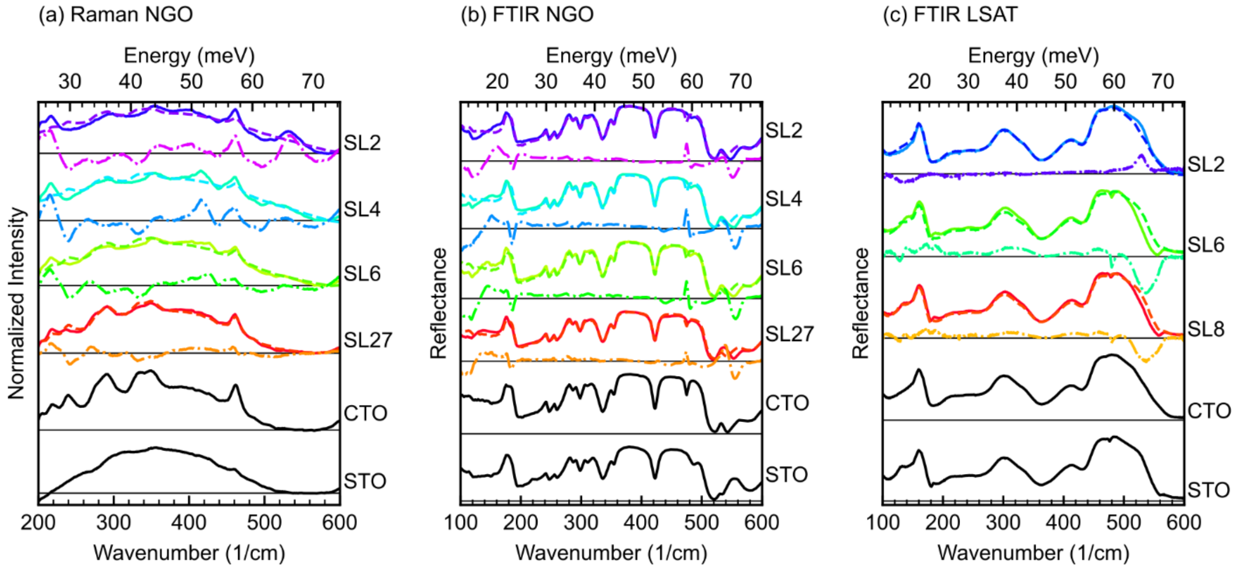

Figure S13. **Interface driven modifications in the macroscopic Raman and infrared response of STO-CTO superlattices.** Raw (solid), fitted (dashed), and residual (dot-dashed) data for (a) UV-Raman and (b) FTIR spectra taken from superlattices on an NGO substrate, and (c) FTIR spectra taken from superlattices on a LSAT substrate. 200 nm STO and CTO thin-films on substrates used to fit superlattice spectra are shown as the bottom of each panel. Difference curves are scaled by a factor of two for clarity.

To accentuate the changes that are unique to the superlattice structure, the residual from a fitted linear combination of STO and CTO on substrates is quantified, as shown by dot-dashed lines in Figure S13. With this approach, emergent Raman responses are observed near 220 cm^{-1} (27 meV) and 450-550 cm^{-1} (60-70 meV) that decrease intensity as the superlattice period is increased (Figure S13(a)). A similar evolution is observed in complementary FTIR measurements (Figure S13(b,c)). These emergent modes are similar in energy to that those observed on locally with EELS and DFT. We therefore conclude they are a consequence of the alternative symmetry existing at the interfaces of the superlattice. As such, their strength necessarily scales with interface density, resulting in the strongest response for the moderate- (SL4) and short-period (SL2) superlattices. Simply put, there are more interfaces or tilts characteristic of an interface in these films and thus the interfacial signal is stronger.

Below we present larger panels of spectra in Figure S13, as shown in Figure S14-13. We include vertical grey dotted lines in Figure S14 at 213, 448, 520, and 732 cm^{-1} where strong first-order Raman features would appear if the substrate was being sampled.[12] Features in the region between 200-400 cm^{-1} have previously been interpreted, for CTO, as resulting from O-Ti-O bending vibrations while features in the region between 400-600 have been assigned as Ti-O_3 torsional modes.[13–15] Due to the large mass of Ca or Sr atoms, vibrational modes involving their motion are not expected to be observed in the region between 200-500 cm^{-1}, but rather would occur at lower frequencies not observed by our experiments. Thus, although we cannot make a concrete assignment to each emergent Raman feature, we can infer that spectral features in this region reflect properties of the Ti-O sublattice.

To understand the presence of interface signal and expand upon the difference method described in the main text, we take the absolute value of the residuals then sum above 400 cm^{-1} (49.6 meV) where the majority of observed changes occurred, as shown in Figure S17. The Raman residual decreases with increasing period thickness meaning that the scattering probability from interface modes decreases. The opposite is seen in the FTIR data, where minima describe more interaction with interface modes.

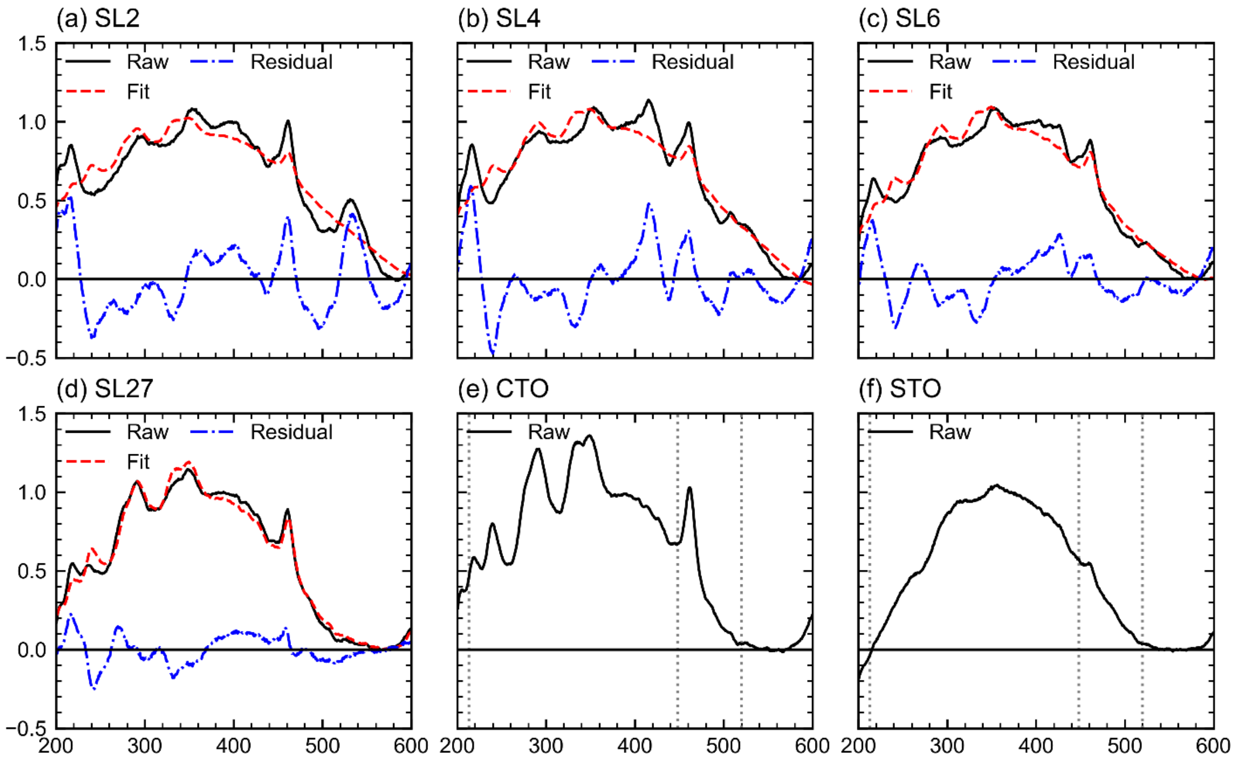

Figure S14. UV-Raman acquired from thin-films on NGO substrates. Grey dotted lines in (e) and (f) indicate energies where first-order NGO Raman peaks would appear if the substrate was being sampled.

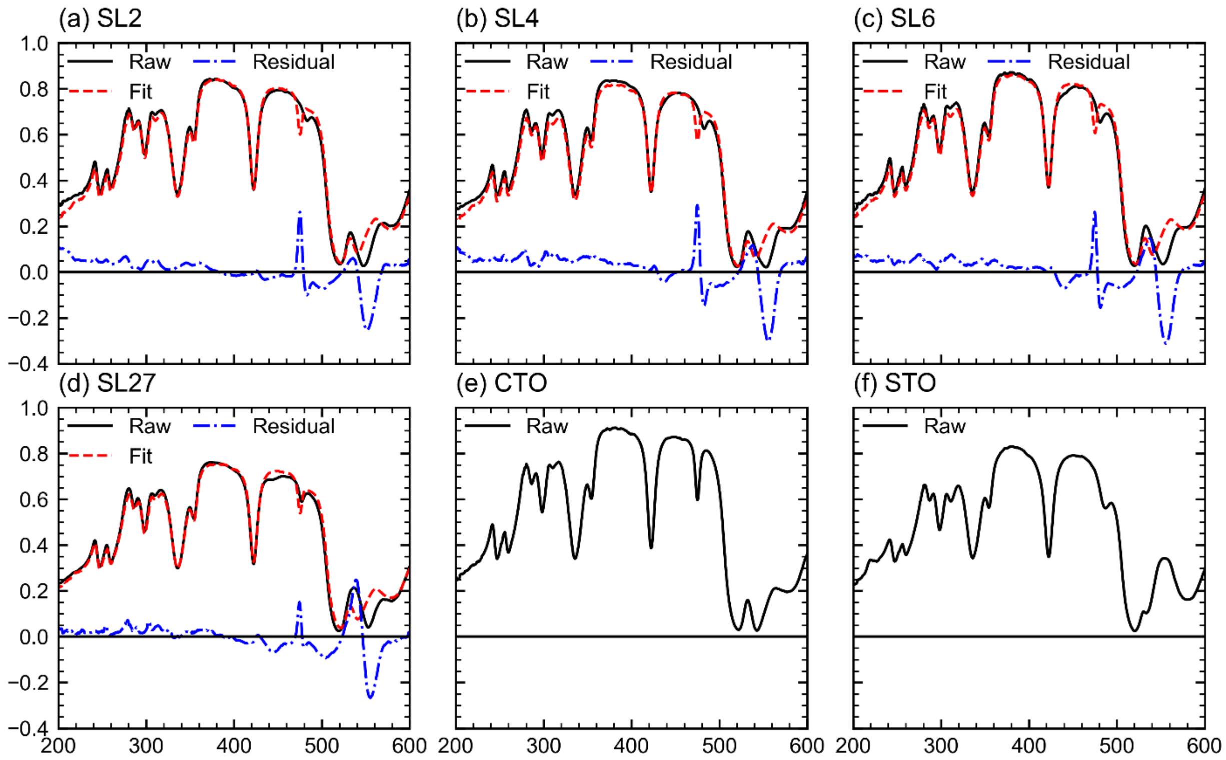

Figure S15. . FTIR acquired from thin-films on NGO.

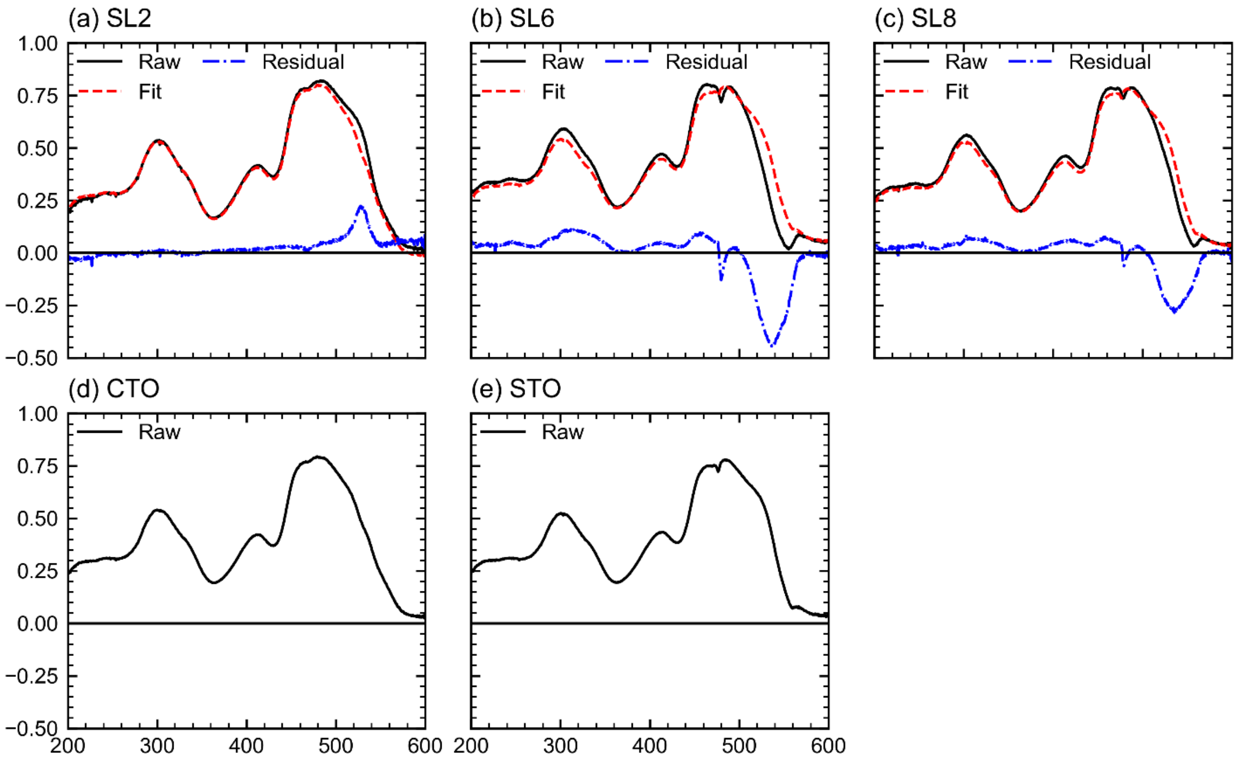

Figure S16. FTIR acquired from thin-films on LSAT.

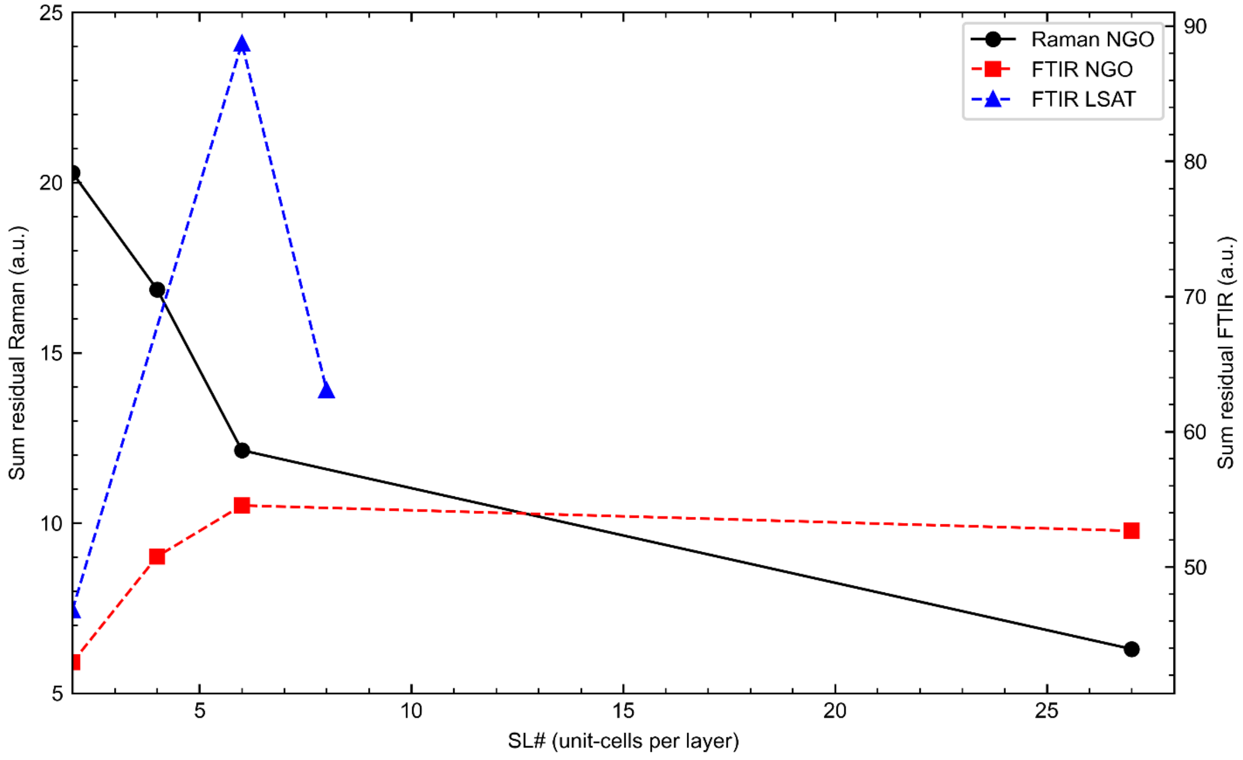

Figure S17. Sum of residuals above 400 cm^{-1} from Raman and FTIR experiments.

S6 Supplemental Information: References

1. Ravichandran, J. et al. Crossover from incoherent to coherent phonon scattering in epitaxial oxide superlattices. Nat. Mater. 13, 168–172 (2014).

2. Hoglund, E. Crystallographic, Electronic, and Phononic Properties of $SrTiO_3$-$CaTiO_3$ Superlattices Versus Layer Thickness. (University of Virginia, 2020-09-30, 2020).

3. Maschek, M. et al. Competing soft phonon modes at the charge-density-wave transitions in DyTe3. Phys. Rev. B 98, 094304 (2018).

4. Naito, M., Ishimaru, M., Hirotsu, Y. & Takashima, M. Identification of soft phonon modes in Ge–Sb–Te using electron diffraction. J. of App. Phys. 98, 034506 (2005).

5. Li, Q. et al. Soft phonon modes and diffuse scattering in $Pb(In_{1/2}Nb_{1/2})O_3$-$Pb(Mg_{1/3}Nb_{2/3})O_3$-$PbTiO_3$ relaxor ferroelectrics. J. of Materiomics 4, 345–352 (2018).

6. Nord, M. et al. Atomic resolution HOLZ-STEM imaging of atom position modulation in oxide heterostructures. Ultramicroscopy 226, 113296 (2021).

7. Nord, M. et al. Three-dimensional subnanoscale imaging of unit cell doubling due to octahedral tilting and cation modulation in strained perovskite thin films. Phys. Rev. Materials 3, 063605–7 (2019).

8. Biegalski, M. D. et al. Impact of symmetry on the ferroelectric properties of $CaTiO_3$ thin films. Appl. Phys. Lett. 106, 162904–15 (2015).

9. Dwij, V. et al. Revisiting 70 years of lattice dynamics of $BaTiO_3$: Combined first principle and experimental investigation. arXiv 16 doi:arXiv:2012.12669.

10. Beechem, T., Graham, S., Hopkins, P. & Norris, P. Role of interface disorder on thermal boundary conductance using a virtual crystal approach. Appl. Phys. Lett. 90, 054104 (2007).

11. Wang, Y., Huang, H. & Ruan, X. Decomposition of coherent and incoherent phonon conduction in superlattices and random multilayers. Phys. Rev. B 90, 165406 (2014).

12. Gasparov, L., Jegorel, T., Loetgering, L., Middey, S. & Chakhalian, J. Thin film substrates from the Raman spectroscopy point of view: Thin film substrates-Raman spectroscopy point of view. J. Raman Spectrosc. 45, 465–469 (2014).

13. Shi, F., Fu, G., Xiao, E.-C. & Li, J. Lattice vibrational characteristics and dielectric properties of pure phase $CaTiO_3$ ceramic. J. Mater. Sci.: Mater. Electron. 31, 18070–18076 (2020).

14. Balachandran, U. & Eror, N. G. Laser-induced Raman scattering in calcium titanate. Solid State Commun. 44, 815–818 (1982).

15. McMillan, P. & Ross, N. The Raman spectra of several orthorhombic calcium oxide perovskites. Phys. Chem. Miner. 16, (1988).
